# Iterative image segmentation of plant roots for high-throughput phenotyping

**DOI:** 10.1038/s41598-022-19754-9

**Published:** 2022-10-04

**Authors:** Kyle Seidenthal, Karim Panjvani, Rahul Chandnani, Leon Kochian, Mark Eramian

**Affiliations:** 1 Department of Computer Science, University of Saskatchewan, 110 Science Place, Saskatoon, SK S7N 5C9 Canada; 2 Global Institute for Food Security, University of Saskatchewan, 421 Downey Road, Saskatoon, SK S7N 4L8 Canada

**Keywords:** Computer science, Plant breeding

## Abstract

Accurate segmentation of root system architecture (RSA) from 2D images is an important step in studying phenotypic traits of root systems. Various approaches to image segmentation exist but many of them are not well suited to the thin and reticulated structures characteristic of root systems. The findings presented here describe an approach to RSA segmentation that takes advantage of the inherent structural properties of the root system, a segmentation network architecture we call ITErRoot. We have also generated a novel 2D root image dataset which utilizes an annotation tool developed for producing high quality ground truth segmentation of root systems. Our approach makes use of an iterative neural network architecture to leverage the thin and highly branched properties of root systems for accurate segmentation. Rigorous analysis of model properties was carried out to obtain a high-quality model for 2D root segmentation. Results show a significant improvement over other recent approaches to root segmentation. Validation results show that the model generalizes to plant species with fine and highly branched RSA’s, and performs particularly well in the presence of non-root objects.

## Introduction

### Background

We are in the post-genomic era of biological investigation where tremendous advances in DNA sequencing are enabling researchers to identify differences in genome sequence that allow us to associate genotypes with phenotypes. This involves identifying significant associations between changes in DNA sequence of individuals in a species with specific biological traits, and then identifying the genes underlying variation in those traits or phenotypes. This is certainly the case in agricultural research where computationally-based genotype $\times$ phenotype analysis is allowing for more rapid and targeted identification of the genes underlying crop traits, and that knowledge is then being used to improve those traits via molecular and digital breeding. One major area of crop phenotyping that has expanded tremendously in recent years is the phenotyping and study of root system architecture (RSA), which is how a plant distributes its root system in three dimensions in the growth environment^1^. Roots are often considered the “hidden half” of plant breeding and plant research as their growth in opaque soil environments make them difficult to study and especially hard for plant breeders to see and quantify root traits of interest^2^.

The ability to phenotype root systems on large numbers of plants (hundred to thousands of plants) in genetic mapping populations for a particular crop species has enabled researchers to show that RSA is a key genetic trait controlling efficient acquisition of water and essential mineral nutrients^3–5^. Thus, much of the RSA phenotyping component of plant breeding has been moved from the field to the lab, with the roots of intact plants grown in transparent media (hydroponics, transparant gels, or, as in this paper, the surface of filter paper pouches) for either 2- or 3-dimensional analysis of RSA (for examples see^1,6,7^). This has been done because there are no comparable techniques for imaging roots in soil without damaging the roots or with the throughput necessary to conduct genetic analysis of root architecture traits which requires phenotyping of roots on thousands of plants.

Although the soil is natural medium to grow plant roots, due to covering of some part of the root system by soil and higher background noise between roots and background, can results in failure to detect the parts of the root system leading to inaccurate root phenotyping. Whereas transparent growing mediums such as hydroponic solution, gel and agar medium can provide higher contrast and reduced background noise and better accuracy to capture the whole root system architecture. Despite the availability of 3D root system phenotyping platform in transparent medium like gel^1^, 2D root phenotyping systems setup in transparent growing medium (^8,9^) is currently preferred due to higher throughput and reduced amount of growth medium required^10^. There are mainly two techniques reported for 2D RSA phenotyping in transparent growing medium. A 2D pouch system techniques is used by growing the plant roots on germination paper inside a plastic pouch that can provide the support for 2D RSA growth^9,11^. Images captured by this technique can be analyzed with various 2D root image analysis software for different sets of root RSA traits such as RootNAV^12^, WhinRhizo (Regent Instrument Inc., Ville de Québec, QC Canada) and DIRT^13^. Another 2D RSA phenotyping system is agar plates or gel plates that are use primarily for Arabidopsis root phenotyping^14^ and images can be analyzed with various software such as EZ Rhizo^15^, myRoot^16^. These image analysis platforms often require image cropping and pre processing before the analysis or all the images from batch are acquired in a restricted frame. Our deep-learning-based method eliminates these steps and segments the root system architecture successfully.

In the current study, plant roots were grown in a “pouch system” which is a Plexiglas box that has a number of layers of germination paper with the top layer consisting of black filter paper on which the root grows, which greatly enhances the contrast between the root and background and improves root image resolution. The germination paper enables the hydroponic nutrient solution in which the bottom portion of the layers of germination paper are placed to be drawn by capillary action into the germination paper, providing water and nutrients for root absorption. The root systems are then digitally photographed using a 2D imaging system for capturing high-resolution images at certain time intervals.

These types of lab-based root phenotyping approaches combined with genome-wide genetic analyses of the root traits have enabled researchers to identify genes, for example, that condition deep rooting in rice (DRO1;^5^). The DRO1 gene was then used via transgenic and marker-assisted breeding approaches to increase rice yields in the field under drought, as the deeper rooting allowed the plants to acquire water deeper in the soil profile. Another example has involved modifying the root system to better acquire the most limiting of the major fertilizer nutrients, phosphorous (P), which tends to be fixed to soil particles and accumulates in the top soil in low P soils. Using the 2D root phenotyping tools employed in this study enabled the phenotyping of a 270 line sorghum association panel which led to the identification of *Pstol1* genes (phosphorus tolerance 1) that altered the root architecture to place more and longer lateral roots in the topsoil and significantly increased sorghum yields on low P soils^17^.

There are tremendous opportunities to improve the speed and accuracy of these types of root imaging platforms using deep learning techniques. Once the root images are acquired, they must be processed in ways to enable accurate quantification of both root growth and RSA traits that accurately describe the shape and distribution of the individual roots. This must be done rapidly, to enable the throughput to image hundreds of root systems per day, as a typical mapping population can consist of 200–400 different varieties for a particular crop species, and with 5-10 replicates per plant with 2 or 3 different time points, requires the imaging of root systems on thousands of plants per experiment. Manual and semi-automatic approaches exist^15,18^, but such approaches are time consuming and can require differing levels of subjective judgement depending on the level of automation. Automated approaches stand to improve the speed and accuracy with which RSA can be studied by making use of deep learning techniques^19,20^. Deep learning utilizes artificial neural networks with a large number of hidden layers and complex internal connections to both learn distinguishing features of images and how those features map to decisions such as labeling an image with an object class identity, or classifying individual image pixels as belonging to a foreground object or background, the latter being an example of image segmentation. The exact nature of the neuron layers and the connections between them within a deep neural network is referred to as the network *architecture*.

Deep learning networks are applicable to certain phases of the RSA analysis pipeline. Briefly, the major steps in the pipeline are:

*Preprocessing/Cropping:* The images are prepared for more efficient processing using operations such as de-noising, or cropping away large areas of the image that do not contain roots to reduce image size and processing time for subsequent steps.
*Segmentation of Roots from Background:* The preprocessed images are analyzed and converted to binary images where the state (on/off) of a pixel indicates whether it is background or root (foreground). This can be challenging due to complexity of root structures, uneven lighting, and air bubbles within the root’s growth medium. There are many types of deep network architectures for segmentation that could be applied in this step.
*Abstract Representation:* The segmented image is transformed into an abstract representation of the root system typically consisting of root segments (possibly annotated with thickness), branching points, and tips. At this step, there is potential for deep learning to be used to construct abstract representations of root systems from the segmented images.
*Phenotypic Trait Extraction:* Mathematical traits are computed from the abstract representation that are used as either direct or proxy root system phenotypes.

In this paper we present a new deep network architecture for performing the segmentation step in the canonical RSA analysis pipeline that compares favourably with comparator deep learning approaches. We begin by reviewing some relevant literature.

### Literature review

Douarre et al. made use of a convolutional neural network (CNN) to extract localized features from patches of an input X-Ray tomography image of roots in soil^21^. Once extracted, these features were passed into a support vector machine (SVM) to classify each pixel as either root or soil. Their model was trained on a dataset of synthetic images which they generated based on the properties of a dataset of X-Ray images, and evaluated on the original X-Ray images. For evaluation, they defined a metric of quality which is the sensitivity and specificity scores of the model multiplied together, where a higher score represents a better segmentation. Due to the unconventional metric which they used for evaluation it is difficult to say how this may compare to other approaches.

*RootNav 2.0* was implemented by Yasrab et al. to perform both the task of classifying root tips and the task of producing a segmentation of the root structure automatically^22^. This was done using an encoder-decoder deep network on which the last layers of output are split into a tip detection path and a segmentation path, such that two different outputs are produced representing the tips and segmented structure of the root system. The benefit of this approach is that any features learned by the network to aid in the detection of root tips can be leveraged to improve segmentation, and vice versa. The output segmentation and root tip points are then used to perform an A$^*$ search algorithm^23^ to determine root paths from tip to source. Once the paths have been determined they can be encoded in the root system markup language (RSML) format for later computations.

Another use of deep learning in root segmentation was conducted by Wang et al.^24^ who developed *SegRoot* which makes use of the *SegNet* deep learning architecture developed by Badrinarayanan et al.^25^. Before training the network, they applied a dilation to binary segmentations of the root images to enhance small features of the root system. Once trained, the network produces a probability matrix for each pixel being a root pixel, which is thresholded such that pixels with a probability 0.99 or higher are considered to be root, to produce a binary mask. The mask is then eroded to undo the implicit dilation learned by the network during training. One issue with this approach is that applying a dilation filter could unintentionally fill small holes in the mask, thus removing important details in the root system. Their model was trained and tested on soybean roots.

The U-Net deep learning architecture^26^ has been applied by Smith et al.^27^ to segment chicory roots from soil in 2D images. The Dice loss function was combined with cross-entropy loss and used for training the network on image patches (sub-images of a main image) containing roots. Comparison with a Frangi filter approach shows significant improvement in segmentation quality.

One general characteristic of RSA is that it is comprised of thin branching segments, which shares properties with structures of interest in other fields. One example of this is segmentation of guidewires, which are used by physicians to treat stenosis in patients and are difficult to see during operation. Guo et al. have modified the U-Net architecture to improve real-time viewing of guidewire tips during operation via segmentation of X-Ray fluoroscopy images^28^. They introduced a new layer block which performs summation rather than concatenation to propagate features between layers of the network.

Kassim et al. combined the U-Net architecture with a random forest tree bagger classifier to optimize segmentation of blood vessels (another example of thin, branching structures) from epifluorescent images^29^. The U-Net is trained on contrast equalized images to produce a regression likelihood map based on the green channel of the input image, which is then filtered by hand-crafted feature filters to train the random forest tree bagger classifier for segmentation. The resulting segmentation then undergoes blob removal as a post-processing step. They achieved relatively small improvements with small increases in Dice score (increase of 0.011), accuracy (increase of 0.013), and sensitivity (increase of 0.046) over an optimized U-Net architecture. This approach makes use of hand crafted features to facilitate the use of the random forests classifier, which will likely need to be updated or changed to be generalized to other applications and may hinder the ability of a single trained model to be used on different datasets within a domain.

Finally, Li et al. have proposed an approach they named *IterNet*, which is a structure made of multiple U-Net structures^30^. The U-Net structures each have their own segmentation outputs and loss function, but they feed the learned features of their last hidden layer to the input of the next U-Net, resulting in a refining process which makes small improvements on the output segmentation of the previous network. This model is used to segment retinal vessels, which have similar structure to root systems. This architecture is particularly well-suited to segmentation of thin structures as the refinement process can help to preserve small details of the structure in the segmentation.

Many of the current approaches to RSA analysis make use of generalized segmentation network approaches to extract RSA from images. Such approaches can produce good segmentations but are not specifically designed for the challenges of segmenting the thin branching structure of root systems. It is important that automated RSA software be able to preserve as much of this structure as possible in order to extract the most accurate phenotyping data possible.

Our proposed solution, based on *ITErRoot*, is a method for high-quality segmentation of root structure from 2D images which builds upon the approach by Li et al.^30^ which they used for segmentation of blood vessels in retinal scans which are thin branching structures branching structures similar to roots. The fully automated nature of our *ITErRoot* model can be scaled to systems with multiple GPUs for increased throughput. The iterative network architecture that *ITErRoot* uses allows for refinement of structures identified by each iteration in the network, making it ideal for retaining details of thin structures and preserving their connectivity. These details are constantly learned during training and are modified at each iteration through the network, which gives a better representation of a general root object rather than a root object specific to one image or species. In addition, we have also developed a novel 2D root image dataset and a tool for consistent annotation of RSA.

## Materials and methods

In this section we outline our approach to the problem of segmenting thin and highly branched root structures. We designed a tool for fast and consistent annotation of root images to produce ground truth binary masks for use in training and evaluation of segmentation algorithms. We also designed a neural network architecture which is particularly suited to segmenting images of roots.

### Image annotation

**Figure 1:**
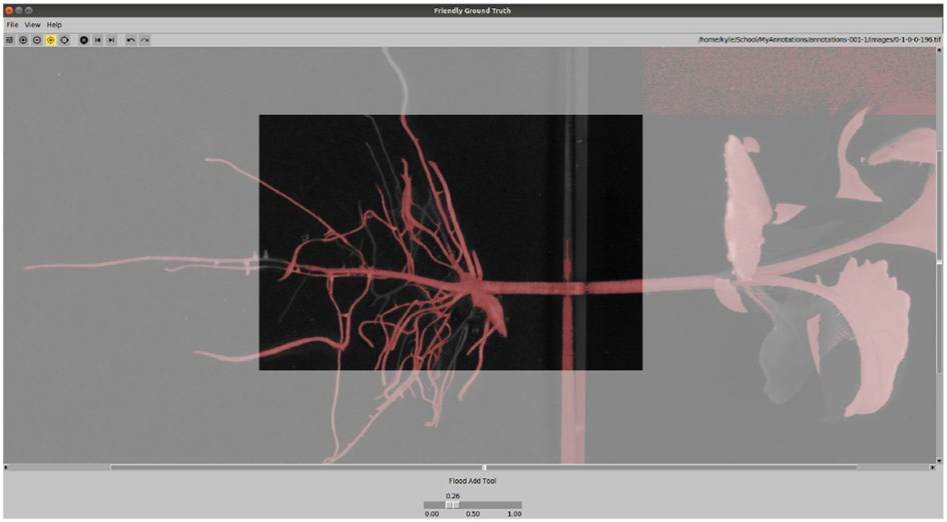
A screenshot of an in-progress annotation on a patch in the *Friendly Ground Truth* tool. Pixels marked as root are indicated to the user in red. Surrounding patches of the current patch are greyed out and not editable, but provide necessary context for the roots which need to be annotated.

In order to train and evaluate our segmentation algorithm we require ground truth segmentation masks with enough detail such that roots in close proximity to each other can be distinguished. To accomplish this task, we designed *Friendly Ground Truth*, an annotation tool which provides a focused view of local root structure for consistent annotation. The tool allows the user to open a large image which is split into smaller non-overlapping patches in a grid formation. The user can then focus on a single patch of the image at a time, making local annotations in greater detail without the distraction of managing the entire image at once. The immediately surrounding patches are visible but greyed out to provide contextual information to the user. The tool provides easy keyboard shortcuts for navigating within and between patches. See Fig. 1 for an example of the tool in use.

Annotation on each patch is carried out using a number of tools: brush tools, localized fill (magic wand) tools, and threshold tools. Brush tools allow the user to use the mouse cursor to draw on the current patch to mark pixels as the foreground class. The radius of the brush can be easily adjusted using the mouse wheel to match the width of a given root, and the brush can be switched to an erase mode to indicate background pixels. Localized fill tools allow the user to select a region to fill with either foreground or background pixels using a threshold. This allows for rapid annotation in regions of similar colour properties to outline foreground and background pixels. The threshold can be easily adjusted using the mouse wheel to grow or shrink the selected region. A global threshold tool is available for quickly applying a threshold to the image, which can be used to annotate large portions of the patch. Once a threshold has been selected, the user can use the tools previously mentioned to make small adjustments where the global threshold does not provide a correct or detailed annotation. Finally, the user has access to a ‘no root tool’ which marks an entire patch as background pixels for cases where a patch does not contain any root. Marking an entire patch this way significantly decreases the amount of time to process an image, as the images contain large areas without foreground; hence, this tool significantly speeds up image processing by allowing a single click method for annotating such patches. When finished, the user has the opportunity to review the image as a whole to see how the individual patch annotations fit together and to make any necessary corrections. The resulting annotation is then exported as a binary mask.

This tool was designed and tested alongside user feedback and suggestions to ensure usability and efficiency in creating annotations. Testing was done by volunteer Computer Science students with experience with other annotation tools. *Friendly Ground Truth* was successfully employed to generate a dataset of root images that were used to train and evaluate the segmentation network structure proposed in this work. The annotation tool has been made publicly available on GitHub (https://github.com/p2irc/friendly_ground_truth) for use by the community to generate root segmentation datasets.

### Iterative neural network architecture

The architecture used in this work is an improvement on the iterative network (IterNET) architecture proposed by Li et al.^30^ called ITErRoot. IterNET consists of a number of refinement networks to iteratively improve the segmentation of the input image. However, the hidden layers of our ITErRoot network are comprised of the residual units introduced by He et al.^31^ which are known to reduce the effects of degrading features as the depth of the network increases. This is different from the architecture used in IterNET which instead used the standard convolutional layers in the U-Net architecture.

**Figure 2:**
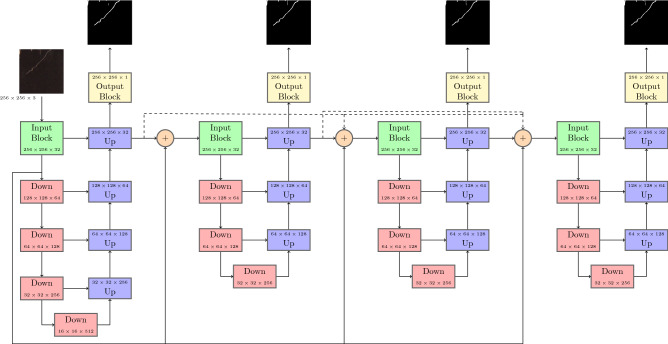
Network architecture diagram showing a high level view of the blocks making up the network. One main U-Net structure acts as input to the subsequent smaller U-Net structure. High- level image features are concatenated to the inputs of all networks via skip connections (solid line), and learned high-level features at the output of each iteration are concatenated together with the inputs of each subsequent iteration (dotted lines). Each network iteration has its own segmentation output, and the last network’s output is considered to be the final segmentation.

**Figure 3:**
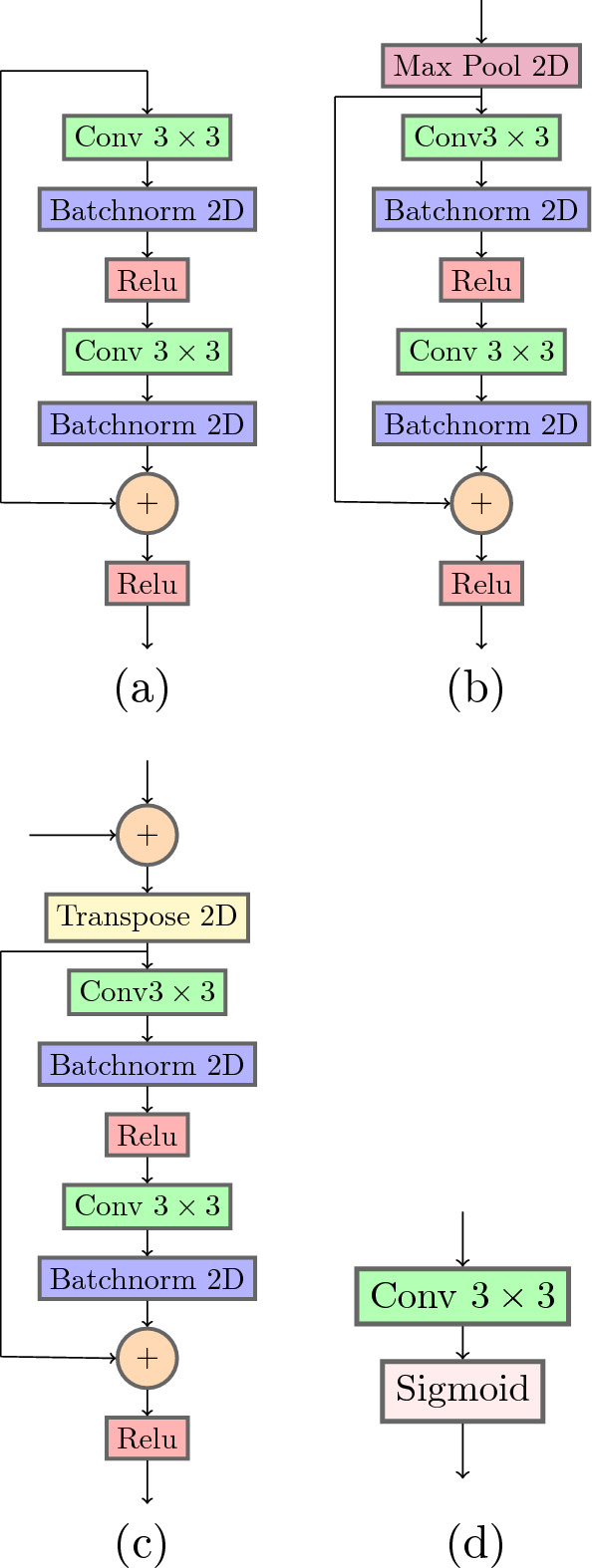
The blocks that make up the network structure. (**a**) The input block based on the residual unit. A $3\times 3$ convolution is applied, followed by batch normalization, and a ReLU function. The same process is repeated again, but the original input features are added together before the last ReLU operation. (**b**)The downward residual block is similar to the input block (**a**) except we apply a 2D max pooling operation to downsample the input features. (**c**) The upward residual block is similar to the input block (**a**) except a transpose is applied to upsample the input features. Features from the corresponding down layer are concatenated before being input to the transpose. (**d**) The output block is a simple $3\times 3$ convolution followed by a sigmoid function to produce a final probability map.

Figure 2 displays a high level view of the architecture used. The architecture is a series of U-Net^26^ structures linked together by hidden and skip connections, where each network is considered to be a new iteration, though in reality it is simply a new network with its own set of learned features. Each iteration has multiple inputs; the output of all previous iterations and the output of the first layer of the main input network, which are concatenated together into a single input tensor. In contrast to the IterNet architecture, we place the concatenation operation before input to each secondary network, rather than after the first input of each secondary network. This allows the secondary networks to incorporate high level details using the entire network while still performing the downsampling operation on the concatenated input. Each U-Net has its own output mask and is evaluated with its own copy of the loss function, but the output of the last hidden layer is used as the input to the next U-Net, in addition to a skip connection from the input layers of the previous two networks. The first network is considered to be the input network, and is made up of four hidden layers. All secondary networks, which come after the input network, are made up of three hidden layers. Each subsequent network after the first is fed a new learned representation of the input image at each epoch as the previous network learns to better represent the desired features for segmentation. This has the effect of refining the segmentation at each iteration to improve small details which are necessary in the segmentation of thin roots in a root system. Another benefit of this architecture is the internal augmentation of training data, as each subsequent network receives a new representation of the input image each epoch, helping to generalize the resulting segmentation for small training datasets. Skip connections from the input layer of previous networks to the input of the current network allow for transmission of high level features from the original input image much in the same way as skip connections in a U-Net architecture preserve high level feature information at each level of the network. The residual units provide skip connections within the hidden layers to preserve high level features as they are propagated through the network, which becomes deeper as the number of iterations increases. Figure 3 details the composition of the blocks used to create the larger network structure.

Rather than using sigmoid cross entropy loss as used by Li et al.^30^, the loss function used for training the architecture is a weighted binary cross entropy combined with Dice loss as defined in Eq. (1). Due to the high imbalance of background and foreground pixels in our images, sigmoid cross entropy loss on its own does not prevent the model from overemphasizing background pixel classification. In Eq. (1a), $\alpha$ and $\beta$ represent the weight of the binary cross entropy and Dice loss functions, respectively. $H(y, {\hat{y}})$, shown in Eq. (1b), represents the binary cross entropy function for some predicted matrix *y* and ground truth matrix ${\hat{y}}$, and $D(y, {\hat{y}})$, shown in Eq. (1c), represents the Dice loss function for the same prediction and ground truth matrices. By allowing each term of the loss function to be weighted as a hyper-parameter, we give greater control over the network during training. This function is applied to each of the networks inside the overall network structure, and the loss for the last iteration is used as the overall loss of the network. The binary cross entropy portion of the loss function pushes the network toward a better per-pixel classification for the image, while the Dice portion characterizes the overall segmentation quality with a standard measure. Early stopping is used to prevent the model from over-fitting and is determined by the tracking the Dice score on the testing set for degradation in performance, and multiplicative learning rate decay is used to help the model converge in later epochs.

1a $$\begin{aligned} Loss= & {} \alpha H(y , {\hat{y}}) + \beta D(y, {\hat{y}}) \end{aligned}$$

1b $$\begin{aligned} H(y, {\hat{y}})= & {} -\sum _{i}^{N}(y_i \log {\hat{y_i}} + (1 - y_i) \log {(1 - \hat{y_i})}) \end{aligned}$$

1c $$\begin{aligned} D(y, {\hat{y}})= & {} \frac{2 \sum _{i}^{N} y_i {\hat{y}}_i}{\sum _{i}^{N} y_i + \sum _{i}^{N} \hat{y_i}} \end{aligned}$$

The network is designed to be used on $256\times 256$-pixel sized image patches to provide enough local information to accurately detect important features, while providing enough context to ensure that features are learned with respect to the overall image. To reduce the probability of over-fitting and increase generalizability of the resulting network we used data augmentation during training. Data augmentation allows us to modify the training images at each epoch, such that for each epoch the network sees a slightly different representation of the data. By using data augmentation, we show the network more possible inputs than are actually present in the static training set of images, forcing the model to learn different representations of the input images and reducing the chances of overfitting the model. We used on-the-fly data augmentation where training images are randomly rotated up to 90 degrees, and/or horizontally and/or vertically flipped. During evaluation, the root images are split into $256\times 256$-pixel overlapping patches with an overlap of 128 pixels (50% of the patch) which ensures that each pixel in the image is examined in four patches. Segmented patches are combined using a majority voting system where a pixel is finally labeled as foreground if it is labeled as foreground in the majority of patches in which it appears. In the case of a tie, the pixel is labelled as background. This helps to improve small errors that may occur due to the way the patches are placed within the full sized input image.

### Datasets

**Figure 4:**
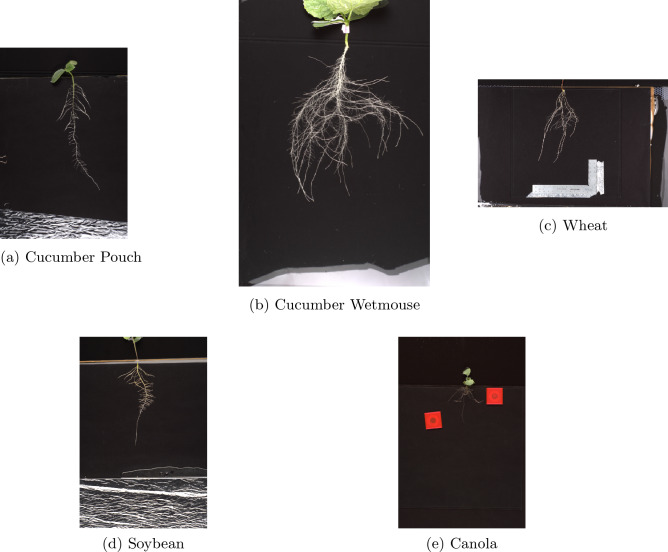
Example images from the Cucumber-Pouch (**a**), Cucumber-Wetmouse (**b**), Wheat (**c**), Soybean (**d**), and Canola (**e**) data sets.

Our overall dataset consists of six separate sets of images of plant roots named: Cucumber-Pouch, Cucumber-Wetmouse, Canola, Wheat, Soybean, and Soybean-assoc. Example images from each set can be seen in Fig. 4. In all of the image sets except Cucumber-Wetmouse, plants were grown in a Plexiglas chamber or “pouch” (first described in "Introduction" section) growing on top of a sheet of black filter paper (to enhance contrast between background and roots), that sits on top of 4 sheets of germination paper that wicks nutrient solution up from the plastic tub in which the bottoms of the pouches are placed during plant growth. The root system on black filter paper is covered with a pliable plastic sheet, which holds the 2D RSA in place and prevents drying of the root system. The root systems are allowed to grow freely in two dimensions, which allows a 2D image to capture the entire root system without requiring a 3D viewpoint. For the Cucumber-Wetmouse image set, the cucumber plants were grown with the roots freely growing in aerated nutrient solution. For imaging, the plant was carefully removed from the hydroponic tub and placed in a shallow glass tray filled with water prior to root system imaging.

Both cucumber sets, the wheat set, and both soybean sets were provided by Dr. Kochian’s research group at the Global Institute for Food Security (GIFS). All five of the image sets were imaged using a Nikon D7200 camera. The Cucumber-Pouch data set consists of forty $4016\times 6016$ images of Cucumber (*Cucumis sativus*) roots taken at 7, 9, and 11 days after transplantation (DAT), the Cucumber-Wetmouse data set consists of thirty nine $4016\times 6016$ images of Cucumber (*Cucumis sativus*) roots imaged at 7, 9, and 11 DAT, the Wheat data set consists of three hundred and one $6016\times 4016$ images of Wheat (*Triticum aestivum*) roots imaged at 7, 9, and 11 DAT, the Soybean data set consists of seventy $6016\times 4016$ images of Soybean (*Glycine max*) roots imaged at 8, 10, and 12 DAT, and the Soybean-assoc data set consists of three hundred thirty three $6016\times 4016$ images of Soybean roots imaged at 5 and 8 DAT.

The Canola image set was provided by Agriculture and Agri-Food Canada (AAFC) and consist of four hundred ninety five $2180\times 2980$ images of Canola (*Brassica napus*), imaged using a Nikon D7200 camera in two different sessions, 4 days apart. All images are RGB colour TIFF images. Table 1 gives a summary of the entire data set.

**Table 1:** Data set summay.

Data set	Species	Image size	Repetitions	Number of images	Total images	Number of ground truthed images
Cucumber-Pouch	*Cucumis sativus*	$4016 \times 6016$	7 DAT	13	40	3
			9 DAT	14		3
			11 DAT	13		5
Cucumber-Wetmouse	*Cucumis sativus*	$4016 \times 6016$	7 DAT	14	39	3
			9 DAT	12		3
			11 DAT	13		1
Wheat	*Triticum aestivum*	$6016 \times 4016$	7 DAT	101	301	10
			9 DAT	100		13
			11 DAT	100		11
Soybean	*Glycine max*	$6016 \times 4016$	8 DAT	24	70	0
			10 DAT	23		3
			12 DAT	23		0
Soybean-assoc	*Glycine max*	$6016 \times 4016$	5 DAT	181	333	20
			8 DAT	152		28
Canola	*Brassica napus*	$2180 \times 2980$	Session 1	249	495	24
			Session 2	243		15
			Session 3	3		0
					1278	142

In the “Repetitions” column, DAT indicates the number of days after transplantation that the imaging took place, while in the case of the canola dataset, each session was taken four days apart, but the number of days afer transplantation is not known.

**Figure 5:**
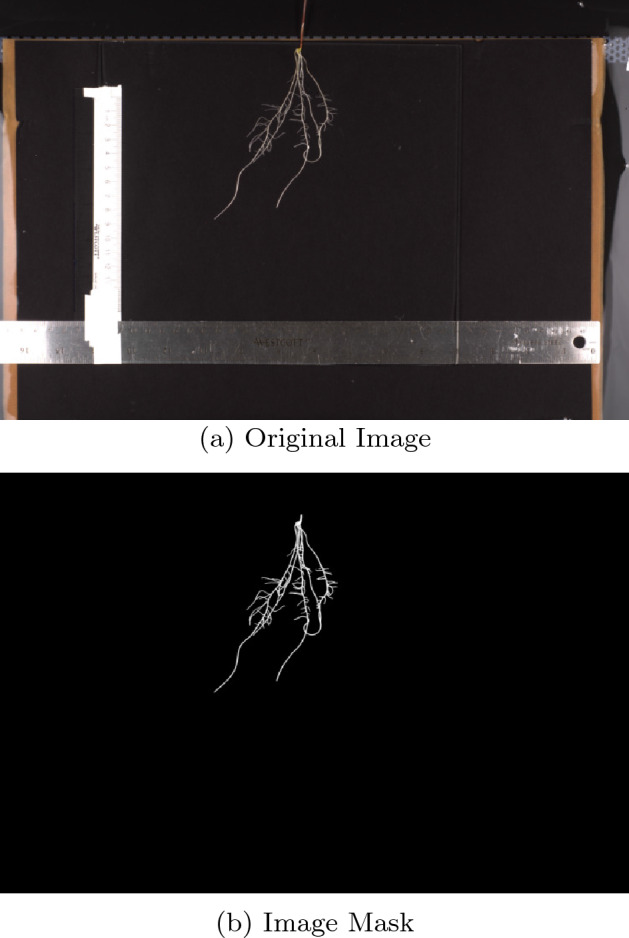
Example image (**a**) with its human annotated ground truth mask (**b**).

Ground truth data was produced using the *Friendly Ground Truth* annotation tool mentioned previously. Annotation was carried out by seven annotators who had little to no previous experience with plant roots, but were experienced with software tools. The annotators were trained to use the tool and were provided with a series of guidelines for annotation to improve consistency among annotators. Due to time constraints only 142 images were able to be annotated. Images were chosen for annotation such that an approximately equal number of images of each species were included. Images from each species were chosen to include different sizes of root systems, as well as varying degrees of overlapping roots to increase the generalizability of the network to a variation of root system sizes and root configurations. Figure 5 gives an example of an image with its annotated mask.

#### Full-image sets

The ground-truthed images were split into a training, testing, and two validation sets. Images containing cucumber root systems were excluded from the training, testing, and first validation set and were placed in their own hold-out second validation set for analyzing the generalizability of the network. The rest of the images were grouped together and placed in training, testing, or validation at random with a 70%-15%-15% split. This generated a final usable dataset consisting of 90 training images, 16 testing images, 18 full validation images, and 18 hold-out cucumber validation images. These sets of images will be referred to as *full-image sets* for the remainder of this paper because they consist of the full-sized images of the entire root systems. These datasets are used in Trial 3 (described below).

#### Derivative datasets

To facilitate thorough analysis of the proposed architecture, we created four additional datasets derived from the full-image set for use with the different trials of the segmentation network architecture we have conducted.

##### Hyper-parameter patch training set

The images in the full-image training set were cropped around the entire root system to reduce the amount of background and then $256\times 256$ size patches were extracted randomly from the cropped images to create a patch training set. This process resulted in 3,282 patches. This hyper-parameter patch training set dataset was used in Trial 1 (described below) to search for training hyperparameters that result in a high-quality trained model.

##### Validation patch set

The *validation patch set* was created from the un-cropped full-image validation set by extracting non-overlapping patches of $256\times 256$ pixels from the full-sized images (not including the hold-out cucumber image set). The full-sized images were not cropped to allow patches containing non-root artifacts so that the ability of the model to correctly label non-root objects could be assessed. This process resulted in 1,864 patches. This was the validation set used for final validation of the model.

##### Testing patch set

The *testing patch set* was created from the full-image testing set using the same process as for the validation patch set, above. This set has 1,077 patches. This was the test set used in Trial 1 and Trial 2 (described below).

##### Expanded patch training set

The second trial we conducted seeks to improve on the results of the first trial (hyper-parameter search) by including additional patches containing only non-root elements that appear in the images. This *expanded patch training set* was created by adding 1,069 $256\times 256$ patches containing no roots to the *hyper-parameter patch training set*. These patches were hand-chosen by a single individual to include image features that the network had difficulty classifying in the first trial, such as the plant stem, water droplets, and dust particles. This dataset was used in Trial 2 (described below).

##### Cropped image set

Finally, it is noted that the full-sized images contain a large amount of area which contains no root system at all. For our fourth trial, we created this *cropped image dataset* which was manually created to ensure that root tips do not appear on the edge of the image. The manual nature of this cropping makes it difficult to specify an amount of pixels used for padding, though a post-cropping analysis shows that a mean number of 153 pixels was allowed for padding. Additionally, the stem of the plant shoot was cropped out of the images so that only roots were visible. This trial aimed to reduce the number of patches that contain non-root objects while also reducing the number of patches that need to be processed to segment a full image. Identically to the full image set, this set is subdivided into testing, validation and hold-out cucumber validation sets.

Table 2 gives a summary of all datasets used for evaluating the network.

**Table 2:** Summary of the datasets used for evaluating the network in different trials.

textbfDataset name	Description	Num images	Trial
Hyper-parameter patch training set	Size $256\times 256$ patches created from the full image set.	3282 Patches	1
Expanded patch training set	Size $256\times 256$ patches created from the full image set, with an additional 1,069 “empty” patches.	4351 Patches	2
Testing patch set	Size $256\times 256$ patches created from the full image set.	1077 Patches	1 & 2
Validation patch set	Size $256\times 256$ patches created from the full image set.	1864 Patches	Validation
Full image set	Full size images including a testing, validation, and hold-out cucumber subset.	16 testing, 18 validation, and 18 cucumber hold-out.	3
Cropped image set	Images from the full image set cropped to various sizes tightly to the root system.	16 testing, 18 validation, and 18 cucumber hold-out.	4

The last column indicates which trial each dataset is used in.

### Segmentation quality metrics

In order to compare and evaluate the quality of one segmentation result to another, a common set of metrics must be used. Herein is used a set of the most common and reliable metrics, namely Dice similarity coefficient (DSC), Intersection over Union (IoU), sensitivity, and specificity.

Segmentation quality metrics are defined in terms of the number of true positives (TP, correctly labeled foreground pixels), true negatives (TN, correctly labeled background pixels), false positives (FP, background pixels incorrectly labeled as foreground), and false negatives (FN, foreground pixels incorrectly labeled as background).

The Dice similarity coefficient (DSC) is defined as

2 $$\begin{aligned} DSC = \frac{2\cdot TP}{2\cdot TP + FP + FN} \end{aligned}$$

A DSC of 0.0 indicates the segmentation is completely disjoint from the ground truth. Higher values of DSC indicate greater overlap with the ground truth, with a DSC of 1.0 indicating perfect segmentation.

IoU is defined as

3 $$\begin{aligned} IoU = \frac{TP}{TP + FP + FN} \end{aligned}$$

IoU quantifies the size of the intersection of the predicted foreground with the ground truth foreground, normalized by the union of their regions. Similarly to Dice coefficient, an IoU score of 0.0 indicates that the segmentation is completely disjoint from the ground truth, while higher values indicate greater overlap with the ground truth. The IoU metric carries a smaller penalty for missed True Positive identification, though is positively correlated with DSC.

Sensitivity is defined as

4 $$\begin{aligned} \mathrm {sensitivity} = \frac{TP}{TP + FN}. \end{aligned}$$

Sensitivity, also known as recall and true positive rate, characterizes how well a model can identify foreground pixels, with 1.0 being a perfect classification of all foreground objects.

Specificity is defined as

5 $$\begin{aligned} \mathrm {specificity} = \frac{TN}{TN + FP}. \end{aligned}$$

Specificity, also known as true negative rate, characterizes how well the model can identify background pixels, with 1.0 indicating a perfect classification of all background pixels.

While accuracy is a common metric used for deep learning applications, it is not suitable for this task. Accuracy is defined as the percentage of pixels that were correctly identified:

6 $$\begin{aligned} \mathrm {accuracy} = \frac{TP + TN}{TP + FP + TN + FN}. \end{aligned}$$

Due to the large number of background pixels in a root image compared to foreground pixels, a model could achieve a very high accuracy score while still misclassifying many of the foreground pixels. For example, if $90\%$ of an image’s pixels are background, one could achieve a $90\%$ accuracy score by simply classifying all pixels as background, which does not make the model any good at identifying roots. For this reason, Dice score is often the most general descriptor of segmentation quality, followed by IoU, sensitivity and specificity. With this in mind, accuracy score is omitted from this work.

### Experimental trials

To evaluate the effectiveness of the proposed model and the effects that inputs have on the quality of output segmentation, we have designed a set of four trials. Preliminary experiments indicated that the ITErRoot architecture with 3 iterations showed the most performance promise, so the larger more time consuming experiments described herein were conducted with a 3-iteration architecture.

#### Trial 1: hyper-parameter tuning

The purpose of this trial was to determine a good set of hyperparameters for training our ITErRoot network architecture. The Google Cloud Platform (cloud.google.com) provides a hyper-parameter tuning framework which allows concurrent training of models with different hyper-parameter inputs. Hyper-parameter inputs are specified as a range of values, and the framework uses Bayesian optimization to search through the space of hyper-parameter values for optimal settings. It will also end a trial early if it proves to be performing worse than previous and concurrent trials in order to conserve computational resources. For this trial we triggered 30 different training jobs within this framework. The input ranges for hyper-parameters are outlined in Table 3. The learning rate and learning rate decay parameters are allowed to fluctuate between a small range of values in order to find an appropriate setting to promote fast convergence at the beginning with slower changes to the network weights as we near the end of training. The binary cross entropy weight in the loss function [$\alpha$ in Eq. (1)] is given a large range to move through in order to allow the search to determine how much it should contribute to the overall segmentation. The weight parameter for the Dice coefficient portion of the loss function [$\beta$ in Eq. (1)] was set to 1.0 to constrain the network to encourage improvement of the Dice score of segmentations no matter the weight assigned to the binary cross entropy factor. Stopping patience, tolerance, and epochs all relate to the early stopping mechanism, where stopping patience is the minimum number of epochs to wait before considering stopping early, stopping epochs is the number of epochs to use to determine whether the result is improving or degrading, and stopping tolerance is a threshold which determines the smallest amount of change in the Dice score in order to stop training: ie. A large stopping tolerance will stop the model even if the Dice score improves by a large margin from the last epoch. A smaller stopping tolerance will allow the model to continue training until there is little change in the Dice score over epoch. The 30 models with varied hyper-parameters were trained on the *hyper-parameter patch training set* and evaluated on the *testing patch set*.

**Table 3:** Input values for hyper-parameter tuning.

Parameter	Type	Min. value	Max. value
Learning rate	Double	0.001	0.01
Learning rate decay	Double	0.85	0.99
Cross entropy weight	Double	0.2	1.0
Stopping patience	Integer	15	20
Stopping tolerance	Double	0.0005	0.01
Stopping epochs	Integer	0	10

#### Trial 2: effects of expanded training set

It is noted that our images contain a large number of non-root objects, such as water droplets, plant shoot, or dust particles. The methods we used for producing the original training set have no way of ensuring that many of these objects appear in the final set of patches. To see if our model performance improves when these objects are intentionally added to the training set, we created the *Expanded Patch Training Set*, which explicitly contains 1, 069 extra patches containing non-root objects. For this trial, we used the highest performing hyper-parameter input values as determined in Trial 1 to re-train the model from scratch on this new data-set. We then directly compare the performance of this new model to the best performing model from Trial 1 on the *Testing Patch Set*.

#### Trial 3: patch-wise segmentation of full images

Our third trial evaluates the model on full sized images from the *Full Image Set* rather than on individual patches. The full images were split into $256\times 256$ patches with an overlap of 128 pixels for the segmentation process to allow the model to process them, and were stitched back together after segmenting the individual patches. The class for each pixel in the overlapping regions was determined by majority vote based on the decided class of the same pixel in each patch it appears in. In the event of a tie, the pixel in question is labelled as background. The model from Trial 2 is directly compared to the best performing model in Trial 1 to determine how the different inputs the models were trained with affect segmentation performance on full sized images.

#### Trial 4: patch-wise segmentation of cropped images

Our fourth and final trial seeks to evaluate the performance of the model on cropped images, which remove as many of the non-root objects from the image as possible without removing parts of the root system. Images from the *Cropped Image Set* are used for this evaluation, and the results of the model from Trial 2 and the best performing model from Trial 1 are compared to determine how the absence of these non-root objects affects the performance of each.

## Results

To thoroughly assess the performance of the proposed network architecture we conducted four experimental trials designed to determine robustness and generalizability, as well as to outline any limitations of our approach. These trials consist of variations of hyper-parameters, the training set, and different types of input images. Models were trained using Google Cloud Platform in a virtual environment with four NVIDIA-Tesla-T4 GPUs. All models were set to train for a maximum of 200 epochs with a batch size of 32. The network was set to use 3 iterations (3 sub networks after the first input U-Net structure), as described above.

### Trial 1: hyper-parameter tuning

The first trial we conducted was designed to determine how hyper-parameter values effect segmentation quality, and to determine the optimal set of hyper-parameter values for the model.

**Table 4:** Model performance for hyper-parameter sets tested during hyper-parameter tuning in descending order of DSC.

DSC	Training steps	Train time	Learning rate	Learning rate decay	Cross-entropy weight	Stopping patience	Stopping tolerence	Stopping epochs
0.936	149	9 hr 28 min	0.00332	0.89661	0.42715	17	0.01000	7
0.934	49	2 hr 29 min	0.00373	0.90361	0.43432	15	0.00995	7
0.926	199	9 hr 39 min	0.00573	0.92774	0.24987	15	0.00932	5
0.925	21	1 hr 14 min	0.00373	0.89465	0.48001	20	0.00995	7
0.924	64	9 hr 33 min	0.00547	0.91167	0.77971	19	0.00533	6
0.922	69	9 hr 40 min	0.00364	0.89527	0.43544	16	0.00711	8
0.921	34	1 hr 51 min	0.00550	0.92000	0.60000	18	0.00525	5
0.919	22	1 hr 18 min	0.00340	0.88736	0.41711	16	0.00614	8
0.918	22	1 hr 15 min	0.00318	0.89646	0.37651	17	0.01000	8
0.918	22	1 hr 15 min	0.00745	0.95032	0.49987	20	0.00319	2
0.917	21	1 hr 13 min	0.00422	0.89580	0.44161	15	0.00738	10
0.916	22	1 hr 13 min	0.00358	0.89545	0.43265	17	0.00702	7
0.916	19	1 hr 6 min	0.00524	0.89901	0.83991	20	0.00456	7
0.911	56	2 hr 53 min	0.00383	0.89582	0.48209	19	0.00995	5
0.911	26	1 hr 26 min	0.00346	0.89518	0.45709	18	0.01000	7
0.908	25	1 hr 24 min	0.00485	0.89783	0.86183	20	0.00375	7
0.906	26	1 hr 25 min	0.00370	0.88605	0.43497	17	0.00715	7
0.905	27	1 hr 30 min	0.00371	0.89533	0.43649	15	0.00710	9
0.905	22	1 hr 14 min	0.00142	0.86066	0.23803	15	0.00946	8
0.904	50	2 hr 34 min	0.00322	0.89422	0.42500	17	0.00691	7
0.902	65	6 hr 42 min	0.00574	0.86168	0.62611	18	0.00483	10
0.899	37	1 hr 57 min	0.00323	0.89977	0.38399	16	0.01000	9
0.898	25	1 hr 25 min	0.00408	0.89623	0.54761	18	0.00992	5
0.898	25	1 hr 25 min	0.00352	0.88279	0.38143	16	0.00607	10
0.898	12	45 min 57 sec	0.00371	0.89213	0.49687	16	0.00663	10
0.895	21	1 hr 10 min	0.00349	0.89031	0.36946	16	0.00654	9
0.894	38	2 hr 2 sec	0.00298	0.89994	0.28756	17	0.01000	8
0.885	11	47 min 29 sec	0.00599	0.93184	0.64064	17	0.00679	7
0.866	7	33 min 24 sec	0.00443	0.91120	0.44106	15	0.00690	2
0.808	4	22 min 43 sec	0.00357	0.88582	0.38293	15	0.00671	8

**Figure 6:**
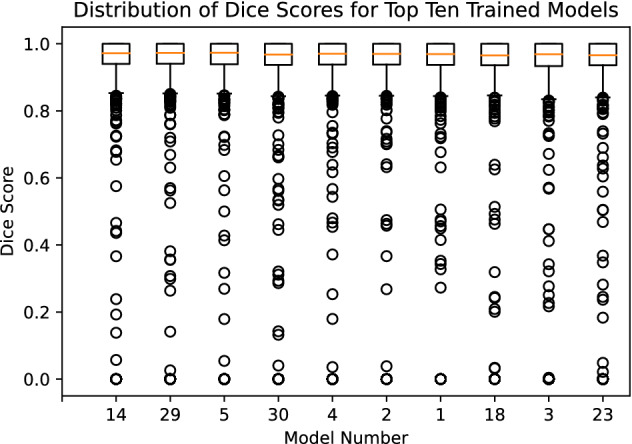
Box plot of the DSC score results for the top ten trained models on the patch testing set in the Hyper-Parameter tuning trial, where the models x-axis are in decreasing order of their mean Dice score. We can see that all models perform quite similarly, with many outliers. These outliers are caused by non-root objects which are present in the input patch.

**Figure 7:**
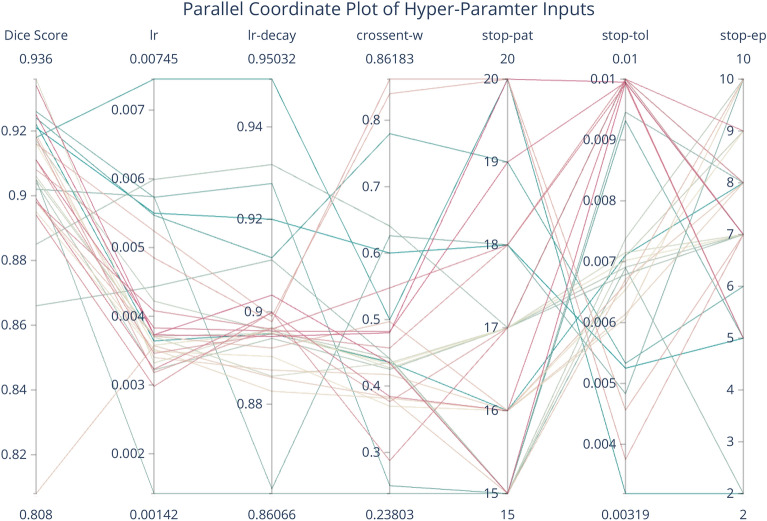
Parallel Coordinate plot describing the relationship between the hyper-parameters and the resulting Dice score for all 30 trials. We can see from this plot that the model prefers a smaller learning rate as well as a larger stopping tolerance. Other parameters seem to have a smaller effect on the overall results.

**Figure 8:**
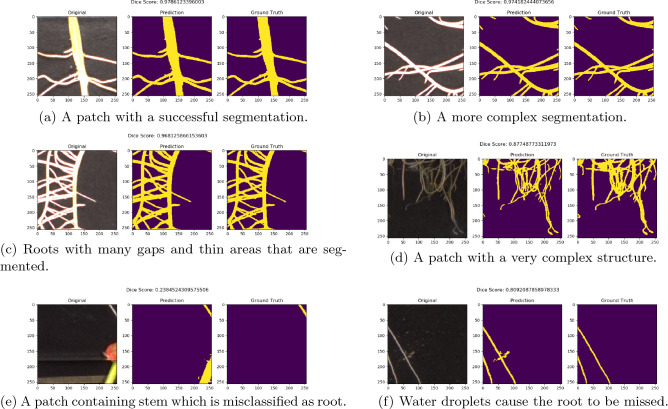
Input, prediction, and ground truth patches for the testing set on the best model found with hyper-parameter tuning. We can see that the root structure is retained with high detail, including fine gaps between roots which grow closely together (**a**)–(**d**). However, when non-root objects, such as plant stem in (**e**) or water droplets in (**f**), are present in the input, we see a degradation of segmentation quality.

**Figure 9:**
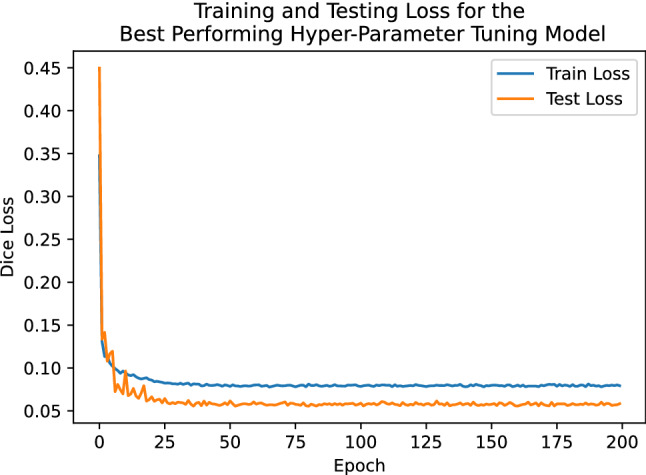
Training and Testing loss of the Hyper-Parameter Tuning model over the course of training. The visible gap between training and testing loss in later epochs may indicate some amount of under-fitting.

Table 4 describes the trained models as evaluated by Dice score on the testing set (see Fig. 6 for the distributions of Dice score for each model). The best-performing trained model resulted in a Dice score of 0.936. A parallel coordinate analysis of these data, shown in Fig. 7, shows that the model performs better with a smaller learning rate and learning rate decay around 0.89. For examples of patch-level predictions, see Fig. 8. We note that the presence of plant stem and water droplets caused misclassification errors. Additionally, Fig. 9 depicts training and testing loss values over the course of training with an indication of possible under-fitting of the dataset.

### Trial 2: effects of expanded training set

**Figure 10:**
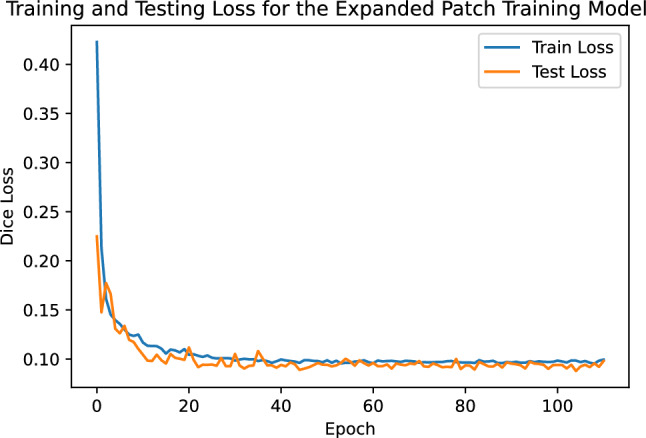
Training and Testing loss of the Expanded Patch model over the course of training. We can see that these curves have a similar trend, with the testing loss being more volatile, while remaining on target with training loss, indicating a good fit of the model.

To address the non-root objects in the full sized images, we re-trained the model with the hyper-parameters from the best performing model from the hyper-parameter tuning trial (Learning Rate: 0.00332, Learning Rate Decay: 0.89661, Binary Cross Entropy Weight: 0.42715, Stopping Tolerance: 0.010, Stopping Epochs: 7, Stopping Patience: 17), this time using the *expanded patch training set*. The *expanded patch training set* contains 1,069 additional hand-chosen patches containing non-root objects with ground truth masks where all pixels are classified as background. The testing set remained as in trial 1. The mean Dice score after re-training was 0.937 with standard deviation 0.160. This is only a marginal difference from the results using the *hyper-parameter patch training set*. A two sample t-test provides no evidence of a statistically significant difference in these results ($p = 0.892$, $\alpha = 0.05$). Figure 10 indicates that the model has achieved a good fit to the data.

### Trial 3: patch-wise segmentation of full images

**Figure 11:**
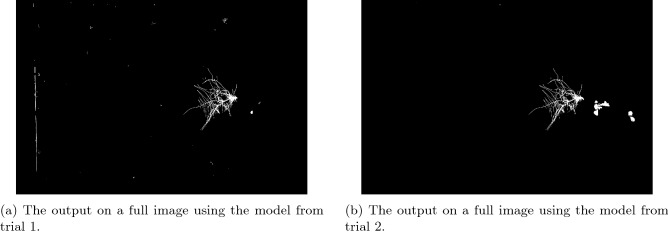
Full image output examples from (**a**) the model trained in trial 1 and (**b**) the model trained in trial 2. The model from trial 1 has many errors around the root system where non-root objects were incorrectly identified as root. The model in trial 2 is more robust to non-root objects in the image giving a much cleaner segmentation, though more of the above-ground plant matter is incorrectly identified as part of the root system.

In addition to evaluating on the *testing patch set*, we evaluated the best trained model from Trial 1 on the *full image testing set*. The final segmented images had mean Dice score of 0.867 with a standard deviation of 0.065. Figure 11a shows an example of a segmented image from the full image testing set. We can see that there are a number of misclassified objects in the image that are not close to the root system.

Similarly, the model from Trial 2 was evaluated on the *full image testing set*. The resulting segmentations had mean Dice score 0.889 with standard deviation 0.070. A two sample t-test comparing the Trial 2 model to the Trial 1 model provides no evidence of a statistically significant difference in these results ($p = 0.377$, $\alpha = 0.05$). However, this model has done a much better job of ignoring non-root objects that are not near the root system, giving a much cleaner segmentation (See Fig. 11b for an example).

### Trial 4: patch-wise segmentation of cropped images

**Figure 12:**
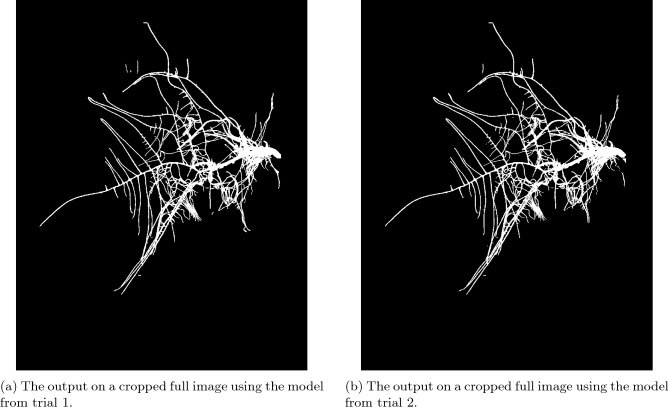
Cropped image output examples from (**a**) the model trained in trial 1 and (**b**) the model trained in trial 2. We can see that when the non-root objects in the image are cropped away that both models give similar segmentations of the root system.

We evaluated the best model from trial 1 and the model from trial 2 on the *cropped image dataset*, which contains the same images from the *full image dataset* hand-cropped to give a tighter focus in the root system. This reduces the amount of non-root patches within the image. The model from Trial 1 gave mean Dice score 0.938 with standard deviation 0.029, while the model from Trial 2 gave a mean Dice score of 0.936 with standard deviation 0.029. Figure 12 shows the resulting segmentations from each model. A two sample t-test shows that there is a statistically significant difference between the results of the *cropped image set* and the *full image set* using the model from trial 1 ($p = 0.001$, $\alpha = 0.05$), and the *full image set* and the *cropped image set* both run on the model from trial 2 ($p = 0.028$, $\alpha = 0.05$). However, there is no evidence of significant difference between the *cropped image set* run on the model from trial 1 and the model from trial 2 ($p = 0.872$, $\alpha = 0.05$). Table 5 gives a summary of the results found in this section.

**Table 5:** Results for each of the models on different testing sets.

textbfTraining set	Testing set	Mean DSC	Mean IoU	Sensitivity	Specificity
Hyper-parameter patch training set	Testing patch set	0.936	0.905	0.963	0.995
	Full image set	0.867	0.771	0.941	0.997
	Cropped image set	0.938	0.884	0.936	0.997
Expanded patch training set	Testing patch set	0.937	0.906	0.950	0.995
	Full image set	0.889	0.807	0.930	0.998
	Cropped image set	0.936	0.881	0.927	0.997

### Validation

After determining that the best model based on the testing data was the model trained on the *expanded patch training set*, it was evaluated on the *validation patch set*, the *full image validation* and *cucumber sets*, and the *cropped image validation* and *cucumber sets*. Results show a mean Dice score of 0.955 with standard deviation 0.149 on the *validation patch set*, mean Dice score of 0.838 with standard deviation 0.235 on the *full image validation set*, and mean Dice score of 0.910 with standard deviation 0.047 on the *cropped validation set*. These results are consistent with what we expect from the results on the testing set. A break down by plant species for the *full image validation set* show a mean Dice score of 0.542 with standard deviation 0.369 on images of canola roots, mean Dice score 0.932 with standard deviation 0.025 on images of soybean roots, and Dice score 0.916 with standard deviation 0.015 on images of wheat roots. A similar breakdown for the *cropped validation set* show mean Dice score of 0.830 with a standard deviation 0.033 on images of canola roots, mean Dice score of 0.953 with a standard deviation 0.012 on images of soybean roots, and a mean Dice score of 0.923 with a standard deviation 0.012 on images of wheat roots. Results from the per-species breakdown are summarized in Table 6. For the hold-out set of cucumber root images the model achieved a mean Dice score of 0.834 with standard deviation 0.123 on the *full image set*, and mean Dice 0.844 with standard deviation 0.131 on the *cropped image set.*

**Table 6:** Per-species breakdown of results on the validation sets.

textbfDataset	Species	Mean DSC	Mean IoU	Sensitivity	Specificity
Full image set	Canola	0.542	0.465	0.813	0.995
	Soybean	0.933	0.875	0.942	0.999
	Wheat	0.916	0.846	0.918	0.995
Cropped image set	Canola	0.830	0.710	0.768	0.993
	Soybean	0.953	0.910	0.950	0.998
	Wheat	0.922	0.855	0.909	0.998

For comparison, we obtained implementations of the *SegRoot* model proposed by Wang et al.^24^ and the *IterNet* model proposed by Li et al.^30^. Both of these models were re-trained from scratch on the *expanded patch training set* and evaluated on each of the *testing patch*, *validation patch*, *full image testing*, *full image validation*, *full image cucumber*, *cropped image testing*, *cropped image validation*, and *cropped image cucumber* sets. For the Full Image Cucumber and Cropped Image cucumber hold-out datasets, we also compare the iterRoot results to thresholding results from winRhizo 2019 (Regent Instruments Inc. Quebec City, Quebec, Canada). Table 7 shows detailed results of our model on each dataset along with results from these comparators.

**Table 7:** Comparison with other models on each dataset.

Dataset	Model	Mean DSC	Mean IoU	Sensitivity	Specificity
Testing patch set	ITErRoot	**0.937**	**0.906**	**0.950**	**0**.**995**
	SegRoot	0.774	0.697	0.789	0.990
	IterNet	0.809	0.760	0.867	0.991
Validation patch set	ITErRoot	**0.955**	**0.936**	**0.963**	**0.997**
	SegRoot	0.836	0.792	0.869	0.992
	IterNet	0.846	0.814	0.903	0.996
Full image testing set	ITErRoot	**0.889**	**0.807**	**0.930**	**0.998**
	SegRoot	0.313	0.194	0.637	0.972
	IterNet	0.399	0.256	0.703	0.972
Full image validation set	ITErRoot	**0.838**	**0.769**	**0.901**	**0.998**
	SegRoot	0.234	0.137	0.640	0.969
	IterNet	0.350	0.222	0.688	0.975
Full image cucumber set	ITErRoot	**0.834**	**0.731**	0.779	**0.998**
	SegRoot	0.426	0.274	0.582	0.970
	IterNet	0.618	0.463	0.703	0.986
	winRhizo-global$^*$	0.368	0.228	0.818	0.932
	winRhizo-local$^*$	0.453	0.296	**0.937**	0.944
Cropped testing set	ITErRoot	**0.936**	**0.881**	**0.927**	0.997
	SegRoot	0.703	0.563	0.647	0.986
	IterNet	0.791	0.687	0.705	**0.999**
Cropped validation set	ITErRoot	**0.910**	**0.838**	**0.889**	0.997
	SegRoot	0.716	0.572	0.639	0.992
	IterNet	0.791	0.674	0.684	**0.999**
Cropped cucumber set	ITErRoot	0.844	0.748	0.779	0.998
	SegRoot	0.720	0.564	0.583	0.998
	IterNet	0.814	0.693	0.708	**0.999**
	winRhizo-global$^*$	0.871	0.782	0.845	0.994
	winRhizo-local$^*$	**0.881**	**0.794**	**0.958**	0. 989

Bold values indicate the best result obtained for each combination of dataset and setmentation quality metric.

All models trained on the training set used in trial 2. $^*$For the Full Image Cucumber Set and the Cropped Cucumber Set, we include threshold-based segmentation with winRhizo. Results are shown for both global and local thresholding (see in "Comparison to segmentation with winRhizo" section).

#### Comparison to segmentation with winRhizo

winRhizo (Regent Instruments Inc., Quebec City, Quebec, Canada) is a widely used commercial software package for segmenting root images and calculating root traits.

For the Full Image Cucumber Dataset, we compared segmentation with winRhizo to segmentation with the ITErRoot model (row 5 of Table 7). We tested winRhizo (2019 version) both in global thresholding mode with an automatically-determined threshold and in local-thresholding mode with automatically-determined thresholds. In global-threholding mode, winRhizo performed worse than all of the deep learning models in terms of mean DSC, mean IoU, and mean specificity but performed better than all of the deep learning models in terms of sensitivity. In local-thresholding mode, winRhizo performed better than in global-thresholding mode for all metrics, and performed worse than the all of the deep learning methods for all metrics except sensitivity, for which it was the top performer.

For the Cropped Image Cucumber Dataset, winRhizo in local thresholding mode performed slightly better than iterRoot in terms of mean DSC, mean IoU, and sensitivity, but was the worst among all methods in terms of specificity. It also outperformed winRhizo in global thresholding mode for all metrics except specificity.

#### Timing results

Table 8 summarizes the time required for ITErROOT prediction and compares the time required to winRhizo. We briefly discuss these results with the understanding that due to licencing of winRhizo, we were not able to time ITErROOT and winRhizo on the same computer.

For cropped images, winRhizo is considerably faster than ITErROOT but it is not much faster than ITErROOT on uncropped images though this lack of difference could be misleading given that the machine we timed ITErRoot on had a faster CPU. We also note that the winRhizo timings also include the time required to compute phenotypes. However, deep learning approaches offer potential for even higher performance given the limited training data that was used in this study, which nonetheless outperformed previous deep-learning approaches.

**Table 8:** Timing results for ITErRoot and winRhizo segementation of the hold-out cucumber datasest.

Dataset	Method	Patch Gen. (s)	Segment. (s)	Reassembling (s)	Total (s)
Full image	ITErRoot	18	69	1.8	89
	winRhizo (global)				70
	winRhizo (local)				46
Cropped Image	ITErRoot	7.7	32	1.1	41
	winRhizo (global)				6.6
	winRhizo (local)				8.4

Results are shown for both the Full Image and Cropped sets. Each set was segmented with ITErRoot, and winRhizo in both global and local thresholding modes. For ITErRoot, times are shown for the patch generation (Patch Gen.), Segmentation (Segment.) and patch reassembling and voting steps. All times per-image averages in seconds.

## Discussion

The results from the hyper-parameter tuning trial give a general outline for good choices about input parameters, but there is no strong evidence indicating that a specific range of values correlate to higher Dice score results. Figure 7 shows us that a smaller learning rate decay value (meaning that the learning rate will decay faster) helps to improve convergence on a better Dice score. Additionally, setting the early stopping metrics to be a bit more conservative seems to give the model more time to approach better results, while still preventing over-fitting of the model. From our experiments, we can see that choice of training data is also an important consideration for improving the robustness of the network to non-root objects.

**Figure 13:**
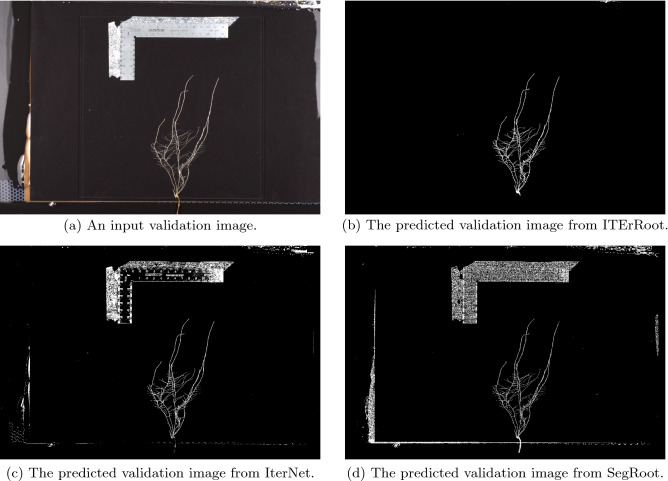
Output segmentations for an input image (**a**) from (**b**) ITErRoot (**c**) IterNet and (**d**) SegRoot. ITErRoot correctly identifies the ruler and surrounding imaging apparatus details as background, while IterNet and SegRoot incorrectly identify these as part of the root system.

Table 7 outlines results from *ITErRoot*, *IterNet*, and *SegRoot* each trained on the training set containing 1,069 extra empty patches from trial 2. We can see that our model excels on each dataset, except in a few cases where the specificity score from *IterNet* is slightly higher (but for those cases the Mean Dice, accuracy and sensitivity scores are still higher for *ITErRoot*). It is important to note that the majority of the pixels in the ground truth masks are background, and so specificity is expected to be high in most cases. Both *IterNet* and *SegRoot* had Dice scores that were similar or better on the cucumber images compared to the validation and test images, while *ITErRoot* saw a small reduction in segmentation quality on the cucumber set. This shows that, while they may not be as good at segmenting roots specifically, these other models are better at more general segmentation of an image. Figure 13 shows an example input image and the resulting segmentations of each of the approaches compared here. The extremely high specificity of ITErRoot relative to comparators likely arises from the IterNET-like structure of our architecture which, as shown by LI et al.^30^, tends to under-segment thin branching structures in the first iteration, and learns how to repair and restore connectivity of those structures in subsequent iterations. This bias towards high specificity is a positive trait for root segmentation since extraneous non-root structures present in the segmentation are likely to be more damaging to the accuracy of subsequent phenotype calculation than missing some actual root structures.

Our experiments show that the network architecture proposed is particularly well suited to segmenting thin root structures, though issues arise in cases where there are no roots present in the input image. It makes sense that the model in trial 2 performed a cleaner segmentation on the full-sized images as it had many specific examples of non-root objects in its training set. We can see that the model in Trial 1 does a good job distinguishing root from other objects, but has trouble identifying non-root objects correctly in the absence of root. We see a slight improvement in the results when annotations of non-root objects are added to the training set. The training set used for the trial 1 model contained patches that did not contain roots, but they were extracted from a cropped region that contained the root system, and so it did not contain a large number of non-root objects. The trial 2 dataset contained patches that specifically focused on non-root objects such as water droplets and dust particles, or edges caused by the imaging apparatus. It is apparent that a better segmentation result can be obtained by removing such objects from the image. Simply cropping the images to reduce the number of these objects can greatly increase segmentation quality. It may also be possible to design image filters which are suited to removing or damping the effect of these non-root objects in the input image, without compromising the information detailing the root system architecture.

**Figure 14:**
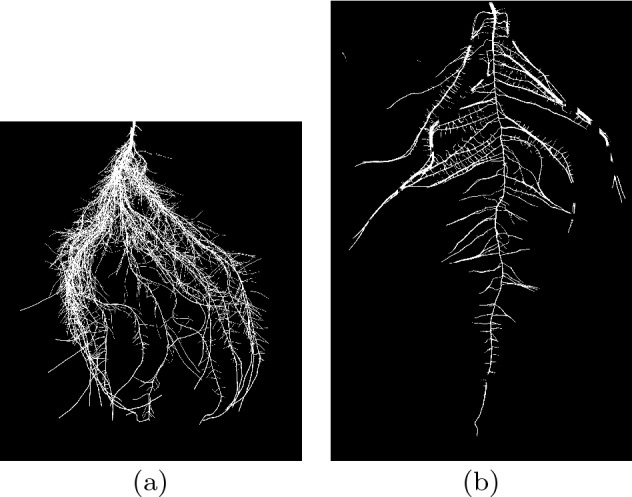
Output segmentation masks from the hold out cucumber set showing (**a**) a segmentation with Dice score 0.9189 and (**b**) a segmentation with Dice score 0.909. Note that in image a) there are many fine details of the root structure which are retained in the segmentation. In image (**b**) we can see that roots which have grown together and appear to be a single larger root contain gaps in their segmentation.

With mean Dice score 0.844 on the *cropped cucumber* set we can see that our model has the potential to generalize to other species of root, with a small drop in segmentation quality. See Fig. 14 for examples of predicted segmentations on the hold-out set of cucumber images. These examples show a segmentation of a cucumber root with many small roots which overlap in several places, and another segmentation where some of the older and longer lateral roots become bundled close together and appear to be one thick root. We can see that in the second image the regions where 2 or more roots are stuck together yield large gaps of misclassified background pixels, resulting in discontinuities in the root system. This suggests that our model could generalize well to root systems with thin roots but begins to degrade when two or more long and thin roots are closely grouped together in the image. This is further backed up by the relatively poor performance on images of canola roots in the validation set, where a number of the lateral roots are clumped together into what are scored as single thicker objects. Images of soybean roots and wheat roots gave better segmentation performance due in part to their more diffusely spread out root systems. Considering our training set, most of the images contained small roots. Many of these images contained plant stems just above the top of the root system, which was annotated as non-root, and so it is possible that the model learned to ignore these large structures, resulting in misclassification of these closely grouped regions of several lateral roots. Fortunately, for roots grown in the pouch system, close grouping of longer lateral roots is a relatively rare occurrence, making our approach ideal for this particular phenotyping setup.

## Conclusion

Segmentation of 2D RSA is an important step in any root phenotyping pipeline. *ITErRoot* offers a fast and accurate method for segmentation of RSA to improve high-throughput phenotyping of roots. Through a rigorous set of trials we have detailed the suitability of *ITErRoot* for learning and identifying root structure, even in complex root systems where traditional methods of image segmentation cannot retain the level of detail necessary for a high-quality segmentation. With proper configuration, the iterative neural network architecture proposed here performs well in the presence of non-root objects, which provides a significant advantage when implementing an automatic high-throughput phenotyping system. Additionally, the *Friendly Ground Truth* tool provides a streamlined process for producing 2D root image datasets for developing and evaluating new approaches to root segmentation, and segmentation of thin structures in general. The contributions of this work not only provide an improvement over comparator deep-learning methods for root segmentation while being competitive with winRhizo, but provides motivation for considering generalizability within the canonical RSA analysis pipeline. Given our significant improvement on other deep learning root segmenetation methods with achieved with limited training data, it is probable that incorporating further training data will continue to improve the model’s accuracy and generalizability to different plant species, eventually unequivocally outperforming local thresholding methods like those used by winRhizo.

As plant breeding trials increase in size and image acquisition systems produce larger datasets of images it will become increasingly important to develop tools which can automatically perform the phenotyping processes necessary for plant breeders to perform their research. Generalizability to a range of different crop species is desirable as it is impractical to develop specific new tools for each new plant species to be studied. This work has explored this concept on the segmentation portion of the RSA pipeline but further work is required to ensure that all processes within the pipeline are fast, accurate, and generalizable to ensure accurate phenotyping results in the face of any plant phenotyping trial.

## Data Availability

The code used to train the neural networks in this study is available on Github (https://github.com/p2irc/ITErRoot). The annotation tool used to create ground truth segmentations for training is available on Github (https://github.com/p2irc/friendly_ground_truth).
